# Silencing Mutant Ataxin-3 Rescues Motor Deficits and Neuropathology in Machado-Joseph Disease Transgenic Mice

**DOI:** 10.1371/journal.pone.0052396

**Published:** 2013-01-22

**Authors:** Clévio Nóbrega, Isabel Nascimento-Ferreira, Isabel Onofre, David Albuquerque, Hirokazu Hirai, Nicole Déglon, Luís Pereira de Almeida

**Affiliations:** 1 CNC - Center for Neurosciences & Cell Biology, University of Coimbra, Coimbra, Portugal; 2 Faculty of Pharmacy, University of Coimbra, Coimbra, Portugal; 3 Faculty of Sciences and Technology, University of Coimbra, Coimbra, Portugal; 4 Department of Neurophysiology, Gunma University Graduate School of Medicine, Maebashi, Gunma, Japan; 5 Lausanne University Hospital, Department of Clinical Neurosciences, Laboratory of Cellular and Molecular Neurotherapies, Lausanne, Switzerland; Dulbecco Telethon Institute and Mario Negri Institute for Pharmacological Research, Italy

## Abstract

Machado-Joseph disease (MJD) or spinocerebellar ataxia type 3 (SCA3) is an autosomal dominantly-inherited neurodegenerative disorder caused by the over-repetition of a CAG codon in the *MJD1* gene. This expansion translates into a polyglutamine tract that confers a toxic gain-of-function to the mutant protein – ataxin-3, leading to neurodegeneration in specific brain regions, with particular severity in the cerebellum. No treatment able to modify the disease progression is available. However, gene silencing by RNA interference has shown promising results. Therefore, in this study we investigated whether lentiviral-mediated allele-specific silencing of the mutant ataxin-3 gene, after disease onset, would rescue the motor behavior deficits and neuropathological features in a severely impaired transgenic mouse model of MJD. For this purpose, we injected lentiviral vectors encoding allele-specific silencing-sequences (shAtx3) into the cerebellum of diseased transgenic mice expressing the targeted C-variant of mutant ataxin-3 present in 70% of MJD patients. This variation permits to discriminate between the wild-type and mutant forms, maintaining the normal function of the wild-type allele and silencing only the mutant form. Quantitative analysis of rotarod performance, footprint and activity patterns revealed significant and robust alleviation of gait, balance (average 3-fold increase of rotarod test time), locomotor and exploratory activity impairments in shAtx3-injected mice, as compared to control ones injected with shGFP. An important improvement of neuropathology was also observed, regarding the number of intranuclear inclusions, calbindin and DARPP-32 immunoreactivity, fluorojade B and Golgi staining and molecular and granular layers thickness. These data demonstrate for the first time the efficacy of gene silencing in blocking the MJD-associated motor-behavior and neuropathological abnormalities after the onset of the disease, supporting the use of this strategy for therapy of MJD.

## Introduction

Machado-Joseph disease (MJD), also designated spinocerebellar ataxia type 3 (SCA3), is the most common dominantly-inherited cerebellar ataxia worldwide [1]–[4]. It is part of a group of nine known polyglutamine (polyQ) disorders which share expanded CAG repeat mutations that translate into polyQ tracts [5], [6]. The signs and symptoms of MJD include progressive postural instability, gait and limb ataxia, weight loss and, in severe cases, premature death [2], [7], [8]. The pathology of MJD includes severe neuronal loss in the spinal cord and selective brain regions such as dentate nuclei (cerebellum), pontine nuclei (brainstem), substantia nigra, and striatum [2], [7]–[10]. MJD is caused by ataxin-3 carrying a stretch of 54–84 consecutive glutamines (mutant ataxin-3) in opposition to normal ataxin-3 whose glutaminic stretch has 14–37 repetitions [11], [12]. The polyQ expansion confers a toxic gain-of-function to the mutant protein, leading to the formation of neuronal intranuclear inclusions, neuronal dysfunction and degeneration [13].

Several therapeutic strategies are under study for MJD treatment such as modulation of Ca2+ signalling, inhibition of calpain-mediated proteolysis of mutant ataxin-3 in the brain or promotion of degradation of mutant ataxin-3 species, either by activation of the proteasome or of the beclin-1 autophagy pathway [14]–[17]. Although such approaches offer promise, the most direct solution to block the pathogenesis of MJD would be to prevent the translation of the mutant ataxin-3 protein in the brain. Accordingly, in conditional transgenic MJD, HD and SCA1 mice shutting off expression of the mutant transgene dramatically slows disease progression and, for selected features, even reverses severe diseases pathology [18], [19].

Decreasing the expression of mutant protein can be achieved by using the RNA interference (RNAi) mechanism to inhibit the expression of the target gene. RNAi-based strategies have been used to supress the expression of toxic polyQ proteins in transgenic mouse models of the polyQ disorders SCA1 and Huntington's disease [20]–[23]. This approach has been refined to discriminate between the wild-type and mutant forms of the messenger RNA by developing silencing sequences targeting single nucleotide polymorphisms (SNP) [24]. In MJD patients, an intragenic single nucleotide polymorphism (SNP) at the 3′ end of the CAG tract of the ataxin-3 gene is present in more than 70% of the cases [25]. This SNP can be used to selectively inactivate mutant ataxin-3, significantly decreasing the severity of the neuropathological abnormalities associated with concomitant induction of MJD, as we previously showed in a (LV)-based rat model of MJD [26]. Nevertheless, no study has ever evaluated the efficacy of gene silencing in a) a transgenic mouse model of MJD, b) exihibiting a severe phenotype, c) regarding motor behavior impairments, d) when initiated after disease onset.

Therefore, in the present study we used lentiviral vectors (LV) encoding short-hairpin RNAs (shRNAs) targeting this SNP, to downregulate mutant ataxin-3 in the cerebellum of a transgenic MJD mouse model that exhibits an early and very severe motor and neuropathological phenotype [15]. This model was generated in a C57/BL6 background, by the introduction of a truncated form of human ataxin-3 with 69 repeats [15], [27] and encoding the C variant of the human ataxin-3 transgene sequence that is present in 70% of the MJD patients [15], [25], [28], making this model particularly adequate for the allele-specific silencing here performed. Transgenic mice expressing a truncated form of the mutant protein have also been used for investigation of gene silencing approaches in Huntington's disease [21], [29]. Moreover, this MJD model displays robust ataxic behavior and neuropathological features from 21 days of age, which makes it time and cost-effective, and mimics a late stage of disease.

In this work we show for the first time that allele-specific silencing of mutant ataxin-3, even when initiated at a late stage of disease dramatically improves motor coordination and reduces neuropathological abnormalities related with MJD.

## Results

### Allele-specific silencing reduces mutant ataxin-3 protein levels and the number of intranuclear inclusions

The polyQ expansion in mutant ataxin-3 confers aggregation properties to the mutant protein, leading to the formation of neuronal intranuclear inclusions, which despite its unclear role in the disease process are a hallmark of MJD [13]. We investigated if specific silencing would reduce the number of aggregates in the cerebellum, a region that is particularly involved in MJD, in a transgenic mouse model of MJD with particular expression of mutant ataxin-3 in Purkinje cells [15]. These transgenic mice were generated using the human ataxin-3 sequence [15] with a polymorphism that is present in 70% of MJD patients [25], [28], which permitted the design of a shRNA that specifically targets mutant ataxin-3 [26]. Therefore, we injected transgenic mice at 3 weeks of age (P21–25) with LV encoding the allele-specific silencing sequence for the human ataxin-3 (shAtx3) or a control sequence (shGFP), see Figure S1. Preliminary experiments in this transgenic model showed that a single injection of 6 µl of lentiviral vectors encoding GFP (*n* = 8) mediated an extensive transduction (around 60%) of the cerebellar cortex (Figure S2 and Figure S3).

**Figure 1:**
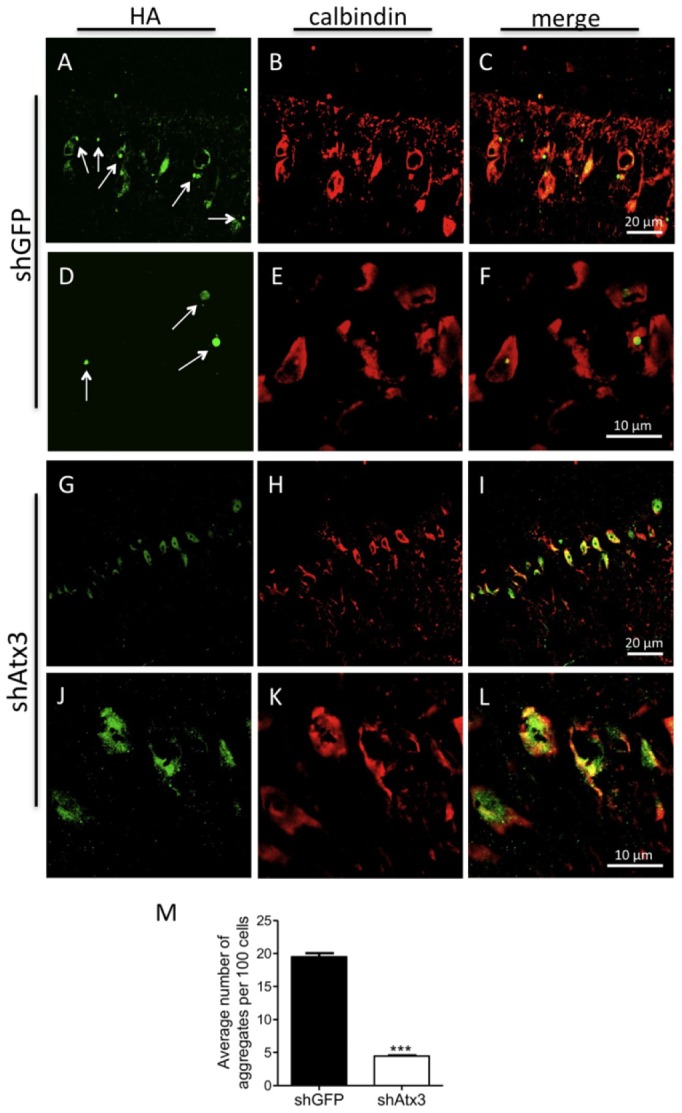
Allele-specific silencing of mutant ataxin-3 reduces the number of intranuclear inclusions. A–L) Confocal analysis of mutant ataxin-3 aggregates in the Purkinje cells at 10 weeks post-injection. Transgenic MJD mice injected at 21–25 days of age with shGFP (control) display intranuclear inclusions of mutant ataxin-3 (revealed by immunohistochemistry with an HA antibody) in Purkinje cells (revealed by calbindin immunohistochemistry) versus a diffuse expression of mutant ataxin-3 in mice injected with the silencing shAtx3. M) The number of intranuclear inclusions is significantly reduced in transgenic mice injected with shAtx3 compared to control animals injected with shGFP (*n* = 6, ***P<0.001; Unpaired Student's *t*-test).

**Figure 2:**
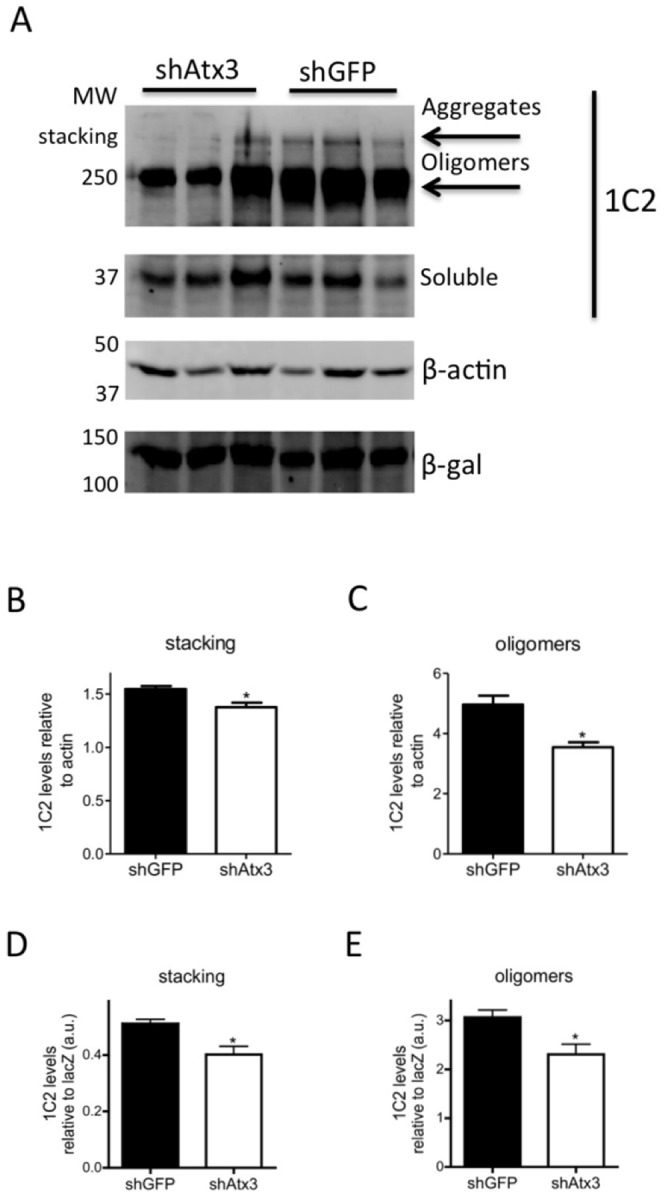
Allele-specific silencing of mutant ataxin-3 reduces the levels of mutant aggregated protein. A) Western blot analysis of cerebellar lysates stained with 1C2 antibody (for the polyglutamine expansion). Differences in levels of high molecular weight protein species were detected between transgenic mice injected with shAtx3 (*n* = 3) and animals injected with shGFP (*n* = 3). Normalization of protein levels was made with β-actin protein endogenous levels, and with β-gal marker (reporter gene of shRNAs lentiviral vector). B, D). The quantification of aggregated protein levels (in the stacking gel) revealed significant differences between transgenic mice injected with shAtx3 and control animals injected with shGFP (*n* = 3; *P<0.05; Unpaired Student's *t*-test). C,E). Oligomerized species were also significantly reduced in animals injected with shAtx3 compared to control animals (*n* = 3; *P<0.05; Unpaired Student's *t*-test).

In the shGFP-injected mice cerebella, we found increased ataxin-3 immunoreactivity in aggregated inclusions (Fig. 1A–F), as compared to shAtx3-injected mice which displayed a diffuse subcellular distribution of ataxin-3 in the cytoplasm of the Purkinje cells (Fig. 1G–L). Importantly, the number of inclusions was robustly and significantly decreased in mice injected with the silencing vectors (shAtx3) as compared to the control ones (*n* = 6; 4.30±0.07 versus 19.5±0.5 nuclear aggregates per 100 Purkinje cells, *P* = 0.0018; Fig. 1M). Further analysis revealed that mice injected with the control vectors encoding the short-hairpin targeting GFP (shGFP) exhibit ataxin-3 aggregates (HA tag) that co-localize with the co-expressed LacZ reporter gene; whereas aggregates from mice injected with vectors encoding the short-hairpin against mutant ataxin-3 (shAtx3) do not co-localize with LacZ and therefore correspond to non-transduced cells (Figure S4). This indicates that expression of the shRNAs against mutant ataxin-3 but not the control sequences prevented formation of ataxin-3 aggregates in the transduced cells. We further investigated the effects of mutant ataxin-3 silencing by western blot analysis of protein levels in the transgenic mice cerebella (Fig. 2A). Probing the membrane with the 1C2 antibody, a marker of the polyglutamine-expanded proteins, revealed a significant decrease in the levels of oligomerized and aggregated ataxin-3 in transgenic mice injected with shAtx3 vectors compared to the control mice injected with shGFP vectors (*n* = 3; aggregates at the upper level of the running and stacking gels Fig. 2B, 2D: 0.44±0.03 versus 0.57±0.03 in control, *P* = 0.0023, normalizing with actin; 0.40±0.02 versus 0.51±0.01 in control, *P* = 0.0028, normalizing with lacZ; and oligomers Fig. 2C, 2E: 3.66±0.17 versus 4.96±0.29 in control, *P* = 0.018 normalizing with actin; 2.32±0.21 versus 3.06±0.14 in control, *P* = 0.04, normalizing with lacZ).

### Silencing of mutant ataxin-3 preserves Purkinje cells integrity and molecular and granular layer thickness in the cerebellar cortex

**Figure 3:**
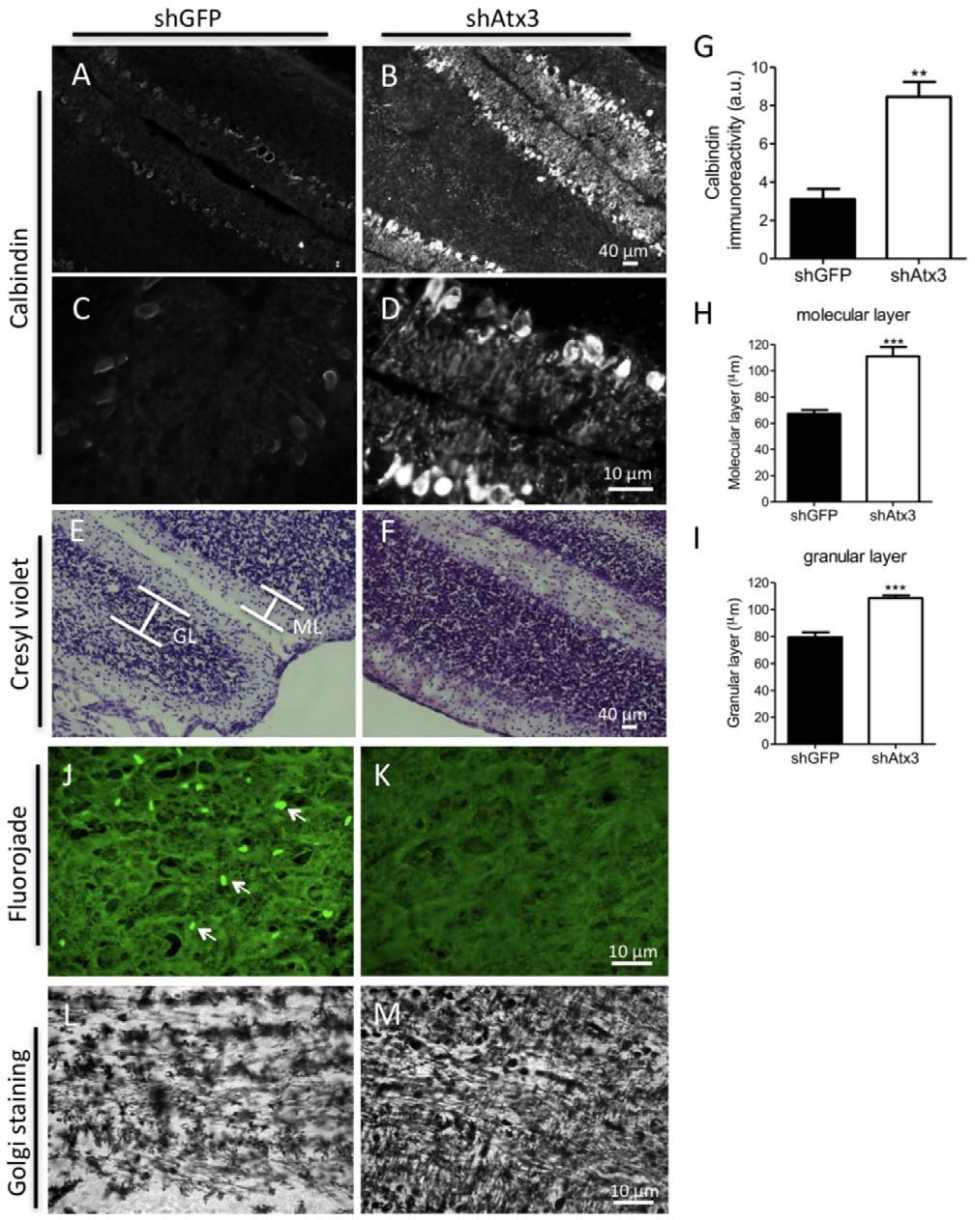
Silencing mutant ataxin-3 reduces Purkinje cell pathology, and prevents neurodegeneration and shrinkage of cellular layers within the cerebellar cortex. A–D) Fluorescence microscopy analysis of calbindin revealed a loss of dentritic arborizations and a shrinkage of Purkinje cells in the mice injected with shGFP (*n* = 8) compared to control mice injected with shAtx3 (*n* = 8). G) Quantification of optical densitometry of calbindin immunoreactivity. Silencing of mutant ataxin-3 preserved immunoreactivity for calbindin in the shAtx3-injected mice as compared to control mice (n = 6; **P<0.01; Unpaired Student's *t*-test). E–F) Cresyl violet staining. A more severe loss of neurons was observed in the control mice (shGFP, *n* = 8) as compared to mice injected with shAtx3. H–I) paralleled by a significant decrease in the size of the molecular (ML) and granular layers (GL) in control mice as compared to shAtx3-injected mice. *Statistical significance (*n* = 6; ***P<0.01; Unpaired Student's *t*-test). J–K) Fluorojade-B staining. Degenerating fluorescently-labelled neurons were only observed in control mice (shGFP, *n* = 8), while no degeneration was observed in mice injected with shAtx3 (*n* = 8). L–M) Golgi staining. A reduction in the number of dentritic arborizations in the molecular layer was observed in mice injected with shGFP (*n* = 8) compared to a higher density of arborizations detected in mice injected with shAtx (*n* = 8). The figure shows representative images that were reproducible among the different groups of animals.

Purkinje cells are key and vulnerable elements regulating the cerebellar function. Therefore we analyzed if silencing mutant ataxin-3 would prevent loss of Purkinje cell markers immunoreactivity and degeneration as well as neurons from molecular and granular layers. Control mice (shGFP) exhibited reduced calbindin immunoreactivity of Purkinje cells (Fig. 3A, C), which was robustly and significantly preserved in mice injected with shAtx3 (Fig. 3B, D, G: *n* = 6; 3.060±0.546 versus 0.852±0.079 in control, *P* = 0.0026). Similarly, silencing mutant ataxin-3 prevented loss of DARPP-32 immunoreactivity (Figure S5).

**Figure 4:**
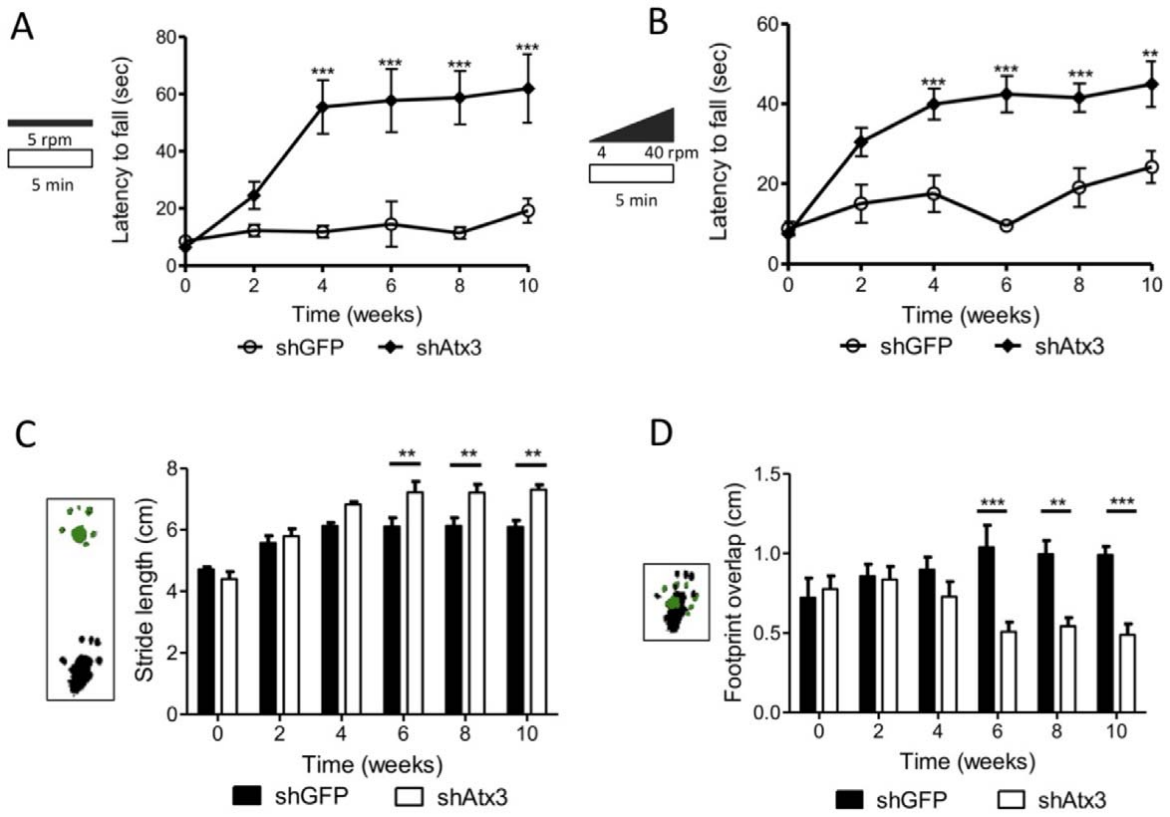
Silencing mutant ataxin-3 improves balance and motor coordination in transgenic MJD mice. A–B) Rotarod performance. Mice injected at 21–25 days of age with shAtx3 (*n* = 8) exhibited a significant better performance in constant velocity (5 r.p.m.) and accelerated rotarod, compared to control mice (shGFP, *n* = 8) from 4 weeks after the injection. C–D) Footprints patterns quantitative analysis, revealed a significantly better motor coordination in mice injected with shAtx3 (*n* = 8) from 6 weeks post-injection compared to control ones (shGFP, *n* = 8), with a higher stride length and a better footprint overlap. *Statistical significance (**P<0.01; ***P<0.001; 2-way ANOVA, Bonferonni post-hoc test).

The cerebellar cortex is characterized by strong cellular interconnectivity, which may propagate the degeneration of one cell to the other. Cresyl violet staining (Fig. 3E, F) revealed that the thickness of the molecular and granular layers were significantly larger in mice injected with shAtx3 as compared to control mice, which suggests a prevention of neurodegeneration (Fig. 3H; molecular layer: *n* = 6; 111.1±7.14 µm versus 67.32±3.08 µm in control, *P* = 0.0005; Fig. 3I; granular layer: *n* = 6; 107.2±2.43 µm versus 77.6±4.42 µm in control, *P* = 0.0004). Furthermore, FluoroJade-B staining, revealed a consistently increased staining suggestive of increased neuronal degeneration in control mice (Fig. 4J), as compared to mice injected with shAtx3 (Fig. 4K). Finnally, Golgi staining revealed reduced arborization in the molecular layer of shGFP-injected mice (Fig. 4L), as compared to mice injected with shAtx3 silencing sequences (Fig. 4M).

### Silencing of mutant ataxin-3 alleviates balance and motor coordination impairments in the MJD transgenic mouse

In order to evaluate whether gene silencing would alleviate gait and limb ataxia the MJD transgenic mice used in this study [15], stereotaxicaly injected with the silencing shAtax3 vectors, were submitted to behavior testing every two weeks until ten weeks post-injection (Figure S6 A). Already at the time of injection, at 21–25 days old, the animals displayed a marked phenotype characterized by an ataxic movement and difficulty to walk and to equilibrate. At the first test performed before the stereotaxic injection, animals barely stayed in the rotarod for 5 seconds, and, throughout the study time course, control mice (injected with shGFP) exhibited drastic impairment in the rotarod task. Silencing of the mutant ataxin-3 robustly enhanced the rotarod performance with mice consistently exhibiting better performances than mice injected with shGFP. These differences were significant and robust from 4 weeks after the injection at constant rotarod velocity (Fig. 4A; *n* = 8; shAtx3 55.5±9.4 s versus shGFP 11.92±2.1 s; *P*<0.001) and in the accelerated test (Fig. 4B; *n* = 8; shAtx3 39.93±3.8 s versus shGFP 17.52±4.59 s; *P*<0.001), and were maintained along the experiment time course (Fig. 4A, B; *n* = 8; 10 weeks; constant: shAtx3 61.95±11.9 s, and shGFP 19.25±4.1 s; accelerated: shAtx3 44.92±5.72 s, and shGFP 24.2±4.02 s). This improvement in motor functions of mice injected with shAtx3 was further investigated by analysis of the footprint patterns. From 4 weeks post-injection mice injected with shAtx3 showed a consistently larger stride length (Fig. 4C) than mice injected with shGFP, a difference that was significant from 6 weeks on (*n* = 8; *P*<0.01), until the end of the experiment at 10 weeks post- injection (*n* = 8; *P*<0.01). A similar pattern and an even more robust effect was registered upon analysis of the footprint overlap (Fig. 4D), where mice injected with shAtx3 revealed a significantly better overlap measure since 6 weeks after the injection compared to control mice (*n* = 8; *P*<0.001). Another quantitative parameter, the hindbase width of footprint patterns confirmed the improved motor performance of shAtx3-injected mice (Figure S6 B).

### Silencing of mutant ataxin-3 enhances locomotor and exploratory activities and reduces anxiety in the MJD transgenic mice

**Figure 5:**
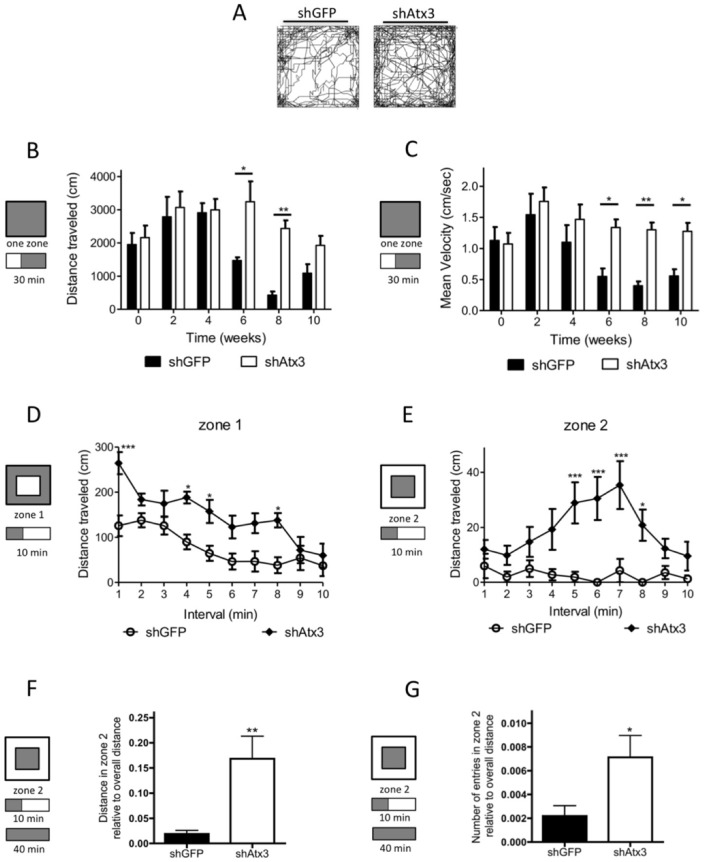
Allele specific silencing of mutant ataxin-3 improves exploratory and locomotor activity and reduces anxiety in MJD transgenic mice. A) Representative plots of moved track at 10 weeks post-injection during 40 minutes in the activity box. Animals injected with shAtx3 (*n* = 8) traveled more that shGFP-injected mice (*n* = 8). The figure shows representative images that were reproducible among the different groups of animals. B–C) Locomotor horizontal activity of mice was tracked for 30 minutes (after a 10 minutes habituation period), and revealed a significantly better locomotor activity of mice injected with shAtx3 (*n* = 8) as compared to control mice (shGFP, *n* = 8), by traveling longer distances with a higher medium velocity. D–E) Two zones analysis of exploratory behavior, revealed that shAtx3-injected mice (*n* = 8) had increased exploratory behavior and were significantly less anxious than controls (shGFP; *n* = 8). F–G) This reduced anxiety of shAtx3-injected mice was confirmed through analyzis of the distance in zone two normalized with the overall distance in the arena, and also the number of entries in zone 2 normalized with the overall distance in the arena. *Statistical significance (*P<0.05; **P<0.01; ***P<0.001; 2-way ANOVA, Bonferonni post-hoc test).

We further analyzed the locomotor activity every two weeks after transduction of mice cerebella by monitoring behavior in an activity box for 30 minutes, after a 10 minutes period of habituation. Animals injected with shAtx3 (*n* = 8) exhibited increased movement as compared to shGFP-injected mice (*n* = 8; Fig. 5A). These differences revealed that animals injected with shAtx3 consistently traveled longer distances than control mice (Fig. 5B; *n* = 8; 6 weeks, *P*<0.05; and 8 weeks, *P*<0.01), exhibited an increase in medium velocity (Fig. 5C), and maximum velocity of movement (Figure S6 C). Analysis of the first 10 minutes in the activity box at 10 weeks post-injection also revealed that mice injected with shAtx3 traveled a longer distance in the first 10 minutes as compared to control mice, revealing an increase in the exploratory behavior (Figure S6 D). Furthermore, when the arena was divided in two zones, it became clear that shAtx3-injected mice traveled a longer distance in zone 1 (Fig. 5D). Also, the control animals (shGFP-injected) traveled to the center of the cage significantly less often than shAtx3-injected mice, suggesting that these latter animals were less anxious (Fig. 5E). In fact, when the total distance or the number of entries in zone 2 was normalized with the total distance, the animals injected with shAtx3 exhibited a less anxious behavior, comparing to control mice shGFP-injected (Fig. 5GF, G).

## Discussion

In this study we show for the first time that allele-specific silencing of mutant ataxin-3 with lentiviral vectors robustly rescues Machado-Joseph disease behavioral and neuropathological phenotype in a severely affected transgenic mouse model.

We previously showed that allele-specific silencing of mutant ataxin-3 expression could mitigate MJD neuropathology induced by viral-mediated expression of mutant ataxin-3 in the rat striatum [26]. In the present work we addressed the question whether this strategy would rescue behavioral and neuropathological impairments, when applied in the cerebellum of a transgenic mouse model already presenting a severe MJD-associated phenotype at the time of viral injection [15]. The transgenic mouse model here used was particularly suited for this purpose as it encoded the human C variant of the human ataxin-3 transgene sequence that is present in 70% of the MJD patients, which allowed allele-specific gene silencing. Moreover, this model encodes a truncated mutant ataxin-3 that induces the strong phenotype that would typically be found in a clinical setting at the time of intervention. Transgenic mouse models expressing truncated forms of the protein have been extensively used to model polyglutamine disorders such as Huntington's disease [21], [29]. In contrast to some of the MJD transgenic mice expressing full-length ataxin-3 developed in the last years [30]–[36], whose behavioral and neuropathological deficits are mild and develop after several months of age, the use of a truncated form of ataxin-3 leads to an early and severe MJD phenotype with robust abnormal neuropathological and behavior deficits while maintaining physiological relevance for this particular question [15], [37].

Gene silencing has been successfully used to downregulate the expression of mutant genes and to rescue phenotypes in various neurodegenerative diseases [21]–[23], [38]–[40]. However, a potential risk of gene silencing lacking discrimination between normal and mutant forms of the causative protein is the loss of the normal protein function. While for some diseases such silencing may not present any drawback [29], for other disorders it is unlikely that the loss of normal protein function would be tolerated. Even though no degenerative phenotype was observed either in a MJD knockout animal [41] or upon non-specific gene silencing in a lentiviral rat model [10], absence of ataxin-3 has been reported to mediate cytoskeletal disorganization and increase cell death in cellular lines [42]. Therefore it may be prudent to either avoid complete silencing of the targeted protein or to employ allele-specific approaches able to preserve the wild-type protein as used in this study. We previously reported that a shRNA allele-specific lentiviral vector permitted selective silencing of mutant ataxin-3 in the striatum, while preserving wild-type ataxin-3, by targeting a C variant of the human ataxin-3 sequence that is present in 70% of the MJD patients [26]. Specific silencing in cellular models has also been reported to SNPs targeting ataxin-7 in SCA7 [43] and huntingtin in Huntington's disease [44], [45]. In the present work, we knocked-down the mutant ataxin-3 transgene carrying the C-polymorphism present in the transgenic animal here used with the previously generated lentiviral vectors encoding an allele-specific shRNA. As there is no homology between mouse ataxin-3 and human ataxin-3 in the region targeted by the silencing sequence (See Fig. S1C), this means that the shRNA used in this study was specific only to human ataxin-3 and therefore does not allow to further infer about the selectivity of the approach, which had been previously demonstrated. Nevertheless, it shows that this allele-specific sequence is highly effective, mediating strong knock-down of the mutant ataxin-3 transgene in the cerebellum and rescuing motor impairments and neuropathology in a severe MJD model, even when initiated at late stage of disease. To our knowledge this is the first demonstration of alleviation of neuropathology and ataxia upon gene transfer of an allele-specific silencing sequence to the mouse cerebellum.

The cerebellum plays an important role in motor coordination and motor learning [46]. Damage of the cerebellum causes motor coordination problems such as gait and limb ataxia that partially explain the disabling clinical features of MJD, as this is one of the most affected regions in the disorder [2], [8]. The MJD transgenic mouse used in this study [15], exhibits mutant ataxin-3 preferential expression in Purkinje cells of the cerebellar cortex, a marked atrophy of the cerebellum already at 3 weeks of age, and pronounced ataxic motor behavior. Similarly, motor incoordination and ataxia development related with progressive Purkinje neuron dendritic and somatic atrophy have been reported in SCA1 transgenic mice [47]. In this study we show that allele-specific silencing of mutant ataxin-3 mediates preservation of dendritic arborizations and Purkinje cells that project into the molecular layer. This may presumably underlie the improved motor performance of animals transduced with shAtx3 as compared to control ones.

The different cell types in the cerebellum are strongly interconnected to each other, and degeneration of one type can lead to atrophy of the interconnected cells. Purkinje cells account for a large fraction of the molecular layer thickness and its atrophy in aging has been associated with a reduction in dendritic arborizations of Purkinje cells [48], [49]. The preservation of Purkinje cells in mice injected with shAtx3 may therefore explain the larger molecular layer thickness in these mice, while Purkinje cell degeneration in control animals probably induces shrinkage of the molecular layer.

Interestingly, in MJD patients the cerebellar cortex and the Purkinje layer are initially relatively preserved, in contrast to other spinocerebellar ataxia such as SCA1 and SCA2 [50]. Instead, neuronal degeneration affects initially dentate nucleus neurons, displaying grumose degeneration [50]. However, as pathology progresses Purkinje cells dysfunction and degeneration develops in MJD mice and patients [51], [52], which may contribute to the retrograde atrophy of cells from molecular and granular layer. Degeneration of Purkinje cells may reduce directly the information input, reduce afferent efficacy, and affect information integration and signal transmission, consequently leading to a reduction in overall cerebellar function. Thus, mutant ataxin-3 silencing in Purkinje cells and other cells in the cerebellar cortex, may prevent the neurodegeneration and cell death in MJD affected regions of the cerebellum. However, translation of this approach to the clinic may have higher chances of success if also targeting the deep cerebellar nuclei, the region with primary degeneration in MJD.

The number of mutant ataxin-3 nuclear inclusions was also significantly reduced in mice injected with shAtx3, as previously observed in the lentiviral-based model [26], in parallel with a subcellular diffuse distribution of the protein in the cytoplasm. The presence of ataxin-3 in the nucleus has been shown to drastically aggravate the pathology in Machado-Joseph disease [32]. Therefore, silencing mutant ataxin-3 may prevent nuclear accumulation and degeneration contributing to preservation of neuronal function and motor behavior of mice injected with shAtx3.

The rotarod test for motor performance is a valid indicator of progressive ataxia; evidentiating proper foot placement and coordination in response to the rotating rod challenges the cerebellum. The allele-specific silencing of mutant ataxin-3 greatly improved the rotarod performance from 4 weeks after the injection, allowing animals to equilibrate in the rotarod for more than 60 seconds, on average 3-fold the time found for control mice. Even though this performance is far from the one that is commonly found in wild-type mice (around 120 s), it corresponds to a very robust preservation of performance. Footprints analysis and activity measures also revealed that mutant ataxin-3 silencing strongly alleviates the phenotype of the MJD transgenic mice. In other polyglutamine disorders silencing of mutant protein also reduced the neurological abnormalities [20]–[22]. Nevertheless, improvement in the rotarod performance upon mutant protein silencing did exceed 0.5 to 1-fold compared to the controls [20], [21].

Our data suggest that shRNA specific silencing reduces the neuropathology and the motor behavior deficits of a severely impaired transgenic MJD mouse model. These results suggest that this approach is a promising therapeutic strategy to MJD patients. Importantly, several questions should be addressed, from the shRNAs long-term safety, to the precise brain regions to be targeted.

## Materials and Methods

### Lentiviral vectors

Viral vectors encoding a short-hairpin targeting the mutant ataxin-3 and the GFP, with the LacZ reporter gene (26) were produced described previously [53].

### In vivo experiments

#### Animals

MJD transgenic mice (C57BL/6 background) expressing the N-terminal-truncated human ataxin-3 with 69 glutamine repeats and an N-terminal hemagglutinin (HA) epitope driven specifically in cerebellar Purkinje cells by the L7 promoter were maintained at CNC by backcrossing heterozygous males with C57BL/6 females [15], [54]. The present study used 8 heterozygous females and 8 heterozygous males injected with lentiviral vectors (in two separate cohorts, with 4 animals per group in each one) encoding the short-hairpin targeting the mutant ataxin-3 (shAtx3; *n* = 8), and as control the short hairpin targeting the GFP (shGFP; *n* = 8). Behavioral testing began at 21–25 days of age (P21–25), and one day after the first behavioral testing animals were submitted to stereotaxic surgery. The experiments were carried out in accordance with the European Community Council directive (86/609/EEC) for the care and use of laboratory animals.

### Stereotaxic injection of lentiviral vectors

The animals (P21–25) received a single 6 µl injection of LV (200 000 ng of p24/ml), at 0.25 µl/min at the coordinates: −1.6 mm rostral to lambda, 0 mm midline, and 1 mm ventral to the skull surface, with the mouth bar set at −3.3.

### Behavioral testing

Mice were trained on a battery of motor tests starting at 21–25 days of age (P21–25) and performed every 2 weeks until 10 weeks by an experienced operator in a blind fashion way. All tests were performed in the same dark room after 30 minutes of acclimatization. Mean values for each measure were calculated and statistical analysis was performed by two-way ANOVA with GraphPad (La Jolla, USA). Data are represented as mean ± SEM.

### Rotarod

Motor coordination and balance were evaluated in a rotarod apparatus (Letica Scientific Instruments, Panlab, Barcelona, Spain). Mice were placed on the rotarod at a constant speed (5 rpm) for a maximum of 5 min, and at accelerated speed (4 to 40 rpm in 5 min) and the latency to fall was recorded. Mice were allowed to perform four trials for each test and time point, with 15 min rest between trials. For analysis, the mean latency to fall off the rotarod of 3–4 trials was used.

### Footprint patterns analysis

The footprint test was used to compare the gait of mice injected with vectors encoding shAtx3 with that of injected with shGFP (control). Hind- and forefeet were coated with black and green nontoxic paints, and the animals allowed walking along a 100-cm-long, 10-cm-wide runaway (with 15-cm high walls) over a fresh sheet of white paper. The footprint patterns were analyzed for (1) stride length, corresponding to the average distance of forward movement between each stride; (2) hind-base width and (3) front-base width measured as the average distance between left and right hind footprints, respectively, and (4) distance from left or right front footprint/hind footprint overlap to measure uniformity of step alternation. The distance between the center of the hind footprint and the center of the preceding front footprint was recorded over a sequence of six consecutive steps, excluding footprints made at the beginning and end of the run. The same operator made all footprints measurements blindly.

### Open field analysis

For the assessment of mice explorative behavior, and locomotor horizontal activity, mice were placed in a 50×50 cm arena with 50 cm high walls and movement activity was recorded for 40 min using Acti-Track System (Panlab, Barcelona, Spain). The collected data was analyzed for the first 10 min, and for the last 30 minutes.

### Histological processing

#### Tissue preparation

Tissue was prepared as previously described (16). Slices throughout the entire cerebellum were collected in superfrost plus microscope slides (Thermo Fisher Scientific, U.S.A) and stored at −20°C before immunohistochemical processing. For each animal the entire cerebellum was collected into 12 slides with 8 coronal sections each, distant 240 µm from each other.

### Immunohistochemical procedure

The immunohistochemical procedure was initiated with a 30 min dehydration at 37°C followed by a 30 min hydration in 0.1 PBS and 1 h blocking in a 0.3% triton in 0.1 PBS with 10% normal goat serum both at room temperature (RT). The following primary antibodies diluted in blocking solution with 0.1% triton were used: mouse monoclonal anti-HA (InvivoGen, San Diego, CA, USA; 1∶1000; O/N, 4°C), and rabbit polyclonal anti-Calbindin D-28K (Chemicon, Temecula, CA, USA; 1∶1000, O/N, 4°C). Sections were then incubated in secondary antibody, goat-anti rabbit and/or mouse conjugated to alexa 488 or 594 (Invitrogen) for 2 h/RT and then mounted in Fluorsave (Calbiochem, Germany) with 4′,6′-diamidino-2-phenylindole (DAPI). Fluorescence images were acquired with a Zeiss Axiovert 200 imaging microscope or LSM Zeiss microscope for double staining experiments.

### Fluorojade B staining

Cerebellar sections were stained with FluoroJade-B (Chemicon, Temecula, CA), an anionic fluorescein derivative that stains neurons undergoind degeneration. The sections were first washed in water and then mounted on silanecoated glass slides, dehydrated, and stained according to the supplier's manual. All photographs for comparison were taken under identical image acquisition conditions and uniform adjustments of brightness and contrast were made to all images.

### Golgi staining

Golgi neurohistological staining was performed following a modification of classical Golgi procedure described previously [55].

### Western blot

Mice cerebella were removed after a sodium pentobarbital overdose, and incubated on ice in a radioimmunoprecipitation assay-buffer solution (50 mM Tris HCl, pH 8, 150 nM NaCl, 1% NP-40, 0.5% sodium deoxycholate, 0.1% sodium dodecyl sulphate) containing proteases inhibitors (Roche diagnostics GmbH) followed by a 4 sec ultra-sound pulse (1 pulse/sec). Total protein lysates were stored at −80°C, protein concentration was determined with the Bradford protein assay (BioRad), and 20 µg of protein extract was resolved in sodium dodecyl sulphate-polyacrylamide gels (4% stacking and 8% running). The proteins were transferred onto polyvinylidene difluoride membranes (GE Healthcare) according to standard protocols. The immunobloting procedure was performed as described previously [10] with the respective primary antibody (1C2, 1∶1000, Millipore), followed by incubation wit the corresponding alkaline phosphatase-linked secondary antibody. Bands were visualized with Enhanced Chemifluorescence substrate (ECF, GE Healthcare) and chemifluorescence imaging (VersaDoc Imaging System Model 3000, Bio-Rad). Membranes were stripped using 0.1 M glycine pH 2.3 (30 min, room temperature) and reprobed with mouse monoclonal anti-β-actin antibody (1∶5000, Sigma), and anti-β-gal antibody (1∶5000, Cell Signaling). Densitometric analysis was carried out in the same gel using Image J software (NIH, USA).

### Quantitative analysis of HA aggregates

Quantification of HA positive inclusions was performed blindly by scanning 4 coronal sections spread over the anterior-posterior extent of the cerebellum of each animal (inter-section distance: 240 µm), using a 20× objective on a Zeiss Axiovert 200 imaging microscope and the image J acquisition and analysis software (NIH, USA). For each coronal section, 8 fields covering the entire cerebellar cortex were digitalized. The total number of HA inclusions, and the number of Purkinje cells were counted, and the average number of inclusions per 100 Purkinje cells was plotted.

### Quantitative analysis of calbindin neuronal expression

Quantification of calbindin immunoreactivity was performed blindly by scanning 4 coronal sections spread over the anterior-posterior extent of the cerebellum (inter-section distance: 240 µm), using a 40× objective on a Zeiss Axiovert 200 imaging microscope. For each coronal section, 8 fields covering the entire cerebellar cortex were digitalized. Optical densitometry analysis was performed with Image J (NIH, USA). Values are represented as the mean value of calbindin optical density per section ± SEM.

### Cresyl violet staining

Cerebellar sections were stained with cresyl violet for 2 minutes, differentiated in acetate buffer pH 3.8 to 4 (2.72% sodium acetate and 1.2% acetic acid; 1∶4 v/v), dehydrated by passing twice through ethanol and toluol solutions, and mounted with Eukitt® (O. Kindler GmbH & CO. Freiburg, Germany).

### Quantification of granular and molecular layers size

Quantification was made over 4 cresyl violet staining coronal sections spread over the anterior-posterior extent of the cerebellum in a blind fashion (inter-section distance: 200–300 µm), using a 20× objective. For each coronal section, 8 fields covering the entire cerebellar cortex were digitalized. For each acquired field at least 6 measurements were made blindly in the same region for all animals, and results converted to µm using Image J software (NIH).

## Supporting Information

Figure S1 A) The transgene used to generate the Q69 transgenic mouse model (Torashima et al., 2008) was isolated from the human ataxin-3 gene with the polymorphism (G→C transition, highlighted in blue) that is present in 70% of MJD patients (Stevanin et al., 1995; Gaspar et al., 1996). B) The presence of this polymorphism permitted the design of an allele-specific silencing of mutant ataxin-3 (Alves et al., 2008), using shRNAs in a LV backbone (with a separate cassette containing the lacZ reporter gene). As control a shRNA targeting GFP in a LV was used. C) There is no homology between mouse ataxin-3 and human ataxin-3 in the region targeted by the silencing sequence, which means that the shRNA used in this study was specific only to human ataxin-3.
(TIF)

Figure S2 Intracerebellar injection of 6 µl (200.000 ng/ml) of lentiviral vectors (LV) encoding for Green Fluorescent Protein (GFP) in the cerebellar vermis of transgenic mice (P21–25) mediates an extensive antero-posterior transduction of the cerebellar cortex (*n* = 8). This extensive transduction was observed from the place of the injection and covered almost 60% of the area of the cerebellar cortex, mainly in the molecular layer and in Purkinje cells.
(TIF)

Figure S3 The intracerebellar injection of LV encoding for GFP in the cerebellar vermis mediates an extensive transduction of the cells in the cerebellar cortex, mainly the molecular layer cells (ML), and the Purkinje layer cells (PCL).
(TIF)

Figure S4 Transgenic mice injected with LV encoding a short-hairpin against GFP (shGFP) exhibit ataxin-3 aggregates (HA tag, green) co-localizing with LacZ (red, white arrows); whereas aggregates from mice injected with short-hairpins against mutant ataxin-3 (shAtx3) do not co-localize. This indicates that the shRNAs targeting mutant ataxin-3, but not the control shRNAs targeting GFP, prevent the formation of ataxin-3 aggregates.
(TIF)

Figure S5 DARPP-32 staining revealed a preservation of immunoreactivity in transgenic mice injected with LV encoding shAtx3 (*n* = 8) as compared to mice injected with shGFP (arrows, *n* = 8). The figure shows representative images that were reproducible among the different groups of animals. Scale bar: 40 µm.
(TIF)

Figure S6 Behavior analysis of studied transgenic mice. A) Time course of mice behavior tests: rotarod performance test, footprints patterns analysis and activity box monitoring were assessed every 2 weeks post-injection until 10 weeks. B) Footprints patterns quantitative analysis. Hindbase width measures of shGFP injected mice (*n* = 8) show a significantly greater distance between left and right limb compared to shAtx3 injected mice (*n* = 8), indicating a higher deficit of coordination in control mice. *Statistical significance (*P<0.05; ***P<0.001; 2-way ANOVA, Bonferonni po*st*-test). C) Locomotor horizontal activity of mice was tracked for 30 minutes (after a 10 minutes habituation period) and analyzed for maximum velocity (cm/sec). Mice injected with shAtx3 (*n* = 8) revealed a significantly better locomotor activity than control mice (shGFP, *n* = 8), as shown by faster movement from 4 weeks post-injection. *Statistical significance (*P<0.05; 2-way ANOVA, Bonferonni *post*-test). D) Allele specific silencing of mutant ataxin-3 improves exploratory activity. Analysis of the first 10 minutes in the cage at 10 weeks post-injection for one zone (arena not divided) revealed significantly-increased traveled distance in mice injected with shAtx3 (*n* = 8) compared to mice injected with shGFP (*n* = 8).
(TIF)
