# Supplementary figures and images for: Silencing Mutant Ataxin-3 Rescues Motor Deficits and Neuropathology in Machado-Joseph Disease Transgenic Mice
Source: PLoS One. 2013 Jan 22;8(1):e52396. doi: 10.1371/journal.pone.0052396 (PMC3551966; doi:10.1371/journal.pone.0052396)

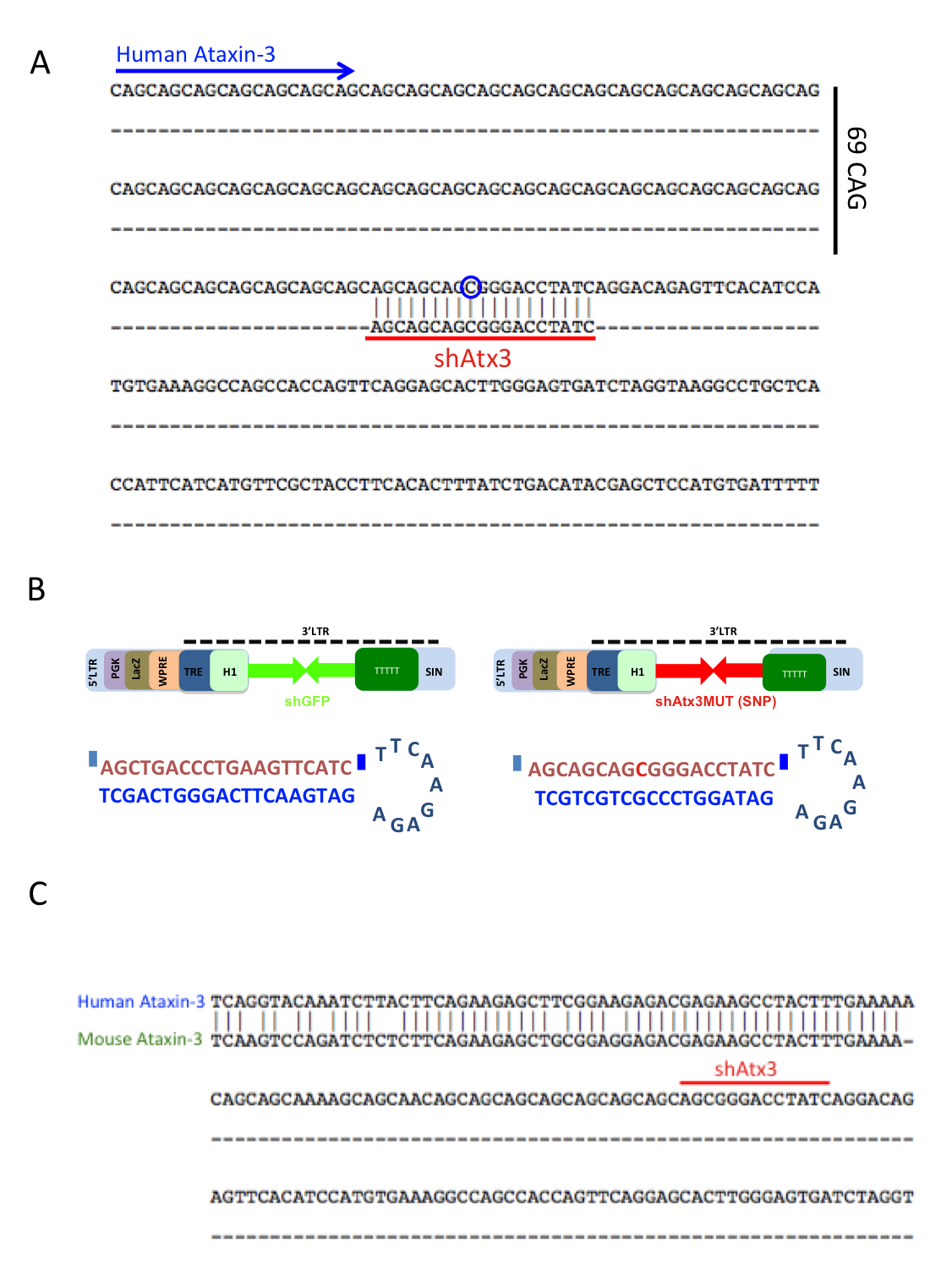

Supplement: Figure S1 — A) The transgene used to generate the Q69 transgenic mouse model (Torashima et al., 2008) was isolated from the human ataxin-3 gene with the polymorphism (G→C transition, highlighted in blue) that is present in 70% of MJD patients (Stevanin et al., 1995; Gaspar et al., 1996). B) The presence of this polymorphism permitted the design of an allele-specific silencing of mutant ataxin-3 (Alves et al., 2008), using shRNAs in a LV backbone (with a separate cassette containing the lacZ reporter gene). As control a shRNA targeting GFP in a LV was used. C) There is no homology between mouse ataxin-3 and human ataxin-3 in the region targeted by the silencing sequence, which means that the shRNA used in this study was specific only to human ataxin-3. (TIF) [file pone.0052396.s001.tif]

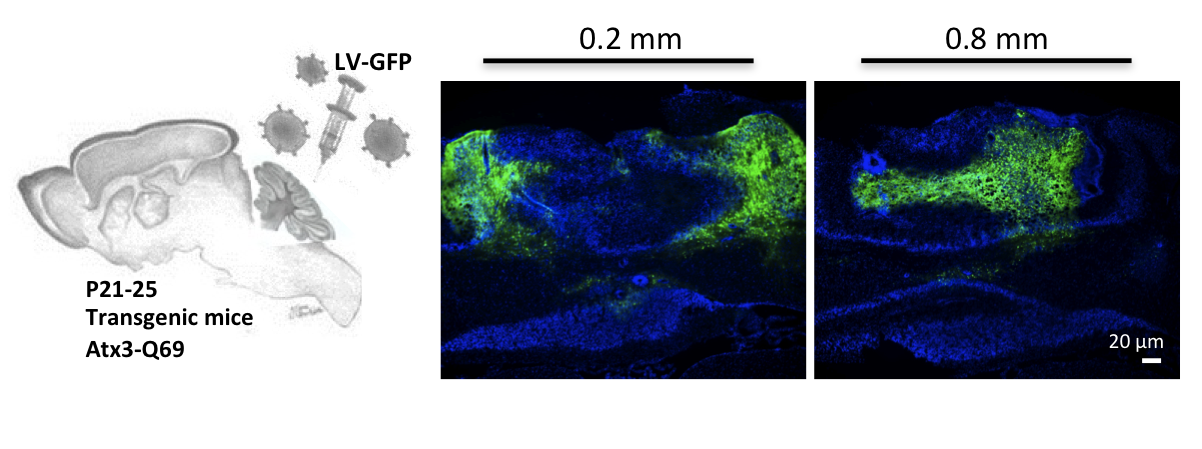

Supplement: Figure S2 — Intracerebellar injection of 6 µl (200.000 ng/ml) of lentiviral vectors (LV) encoding for Green Fluorescent Protein (GFP) in the cerebellar vermis of transgenic mice (P21–25) mediates an extensive antero-posterior transduction of the cerebellar cortex (n = 8). This extensive transduction was observed from the place of the injection and covered almost 60% of the area of the cerebellar cortex, mainly in the molecular layer and in Purkinje cells. (TIF) [file pone.0052396.s002.tif]

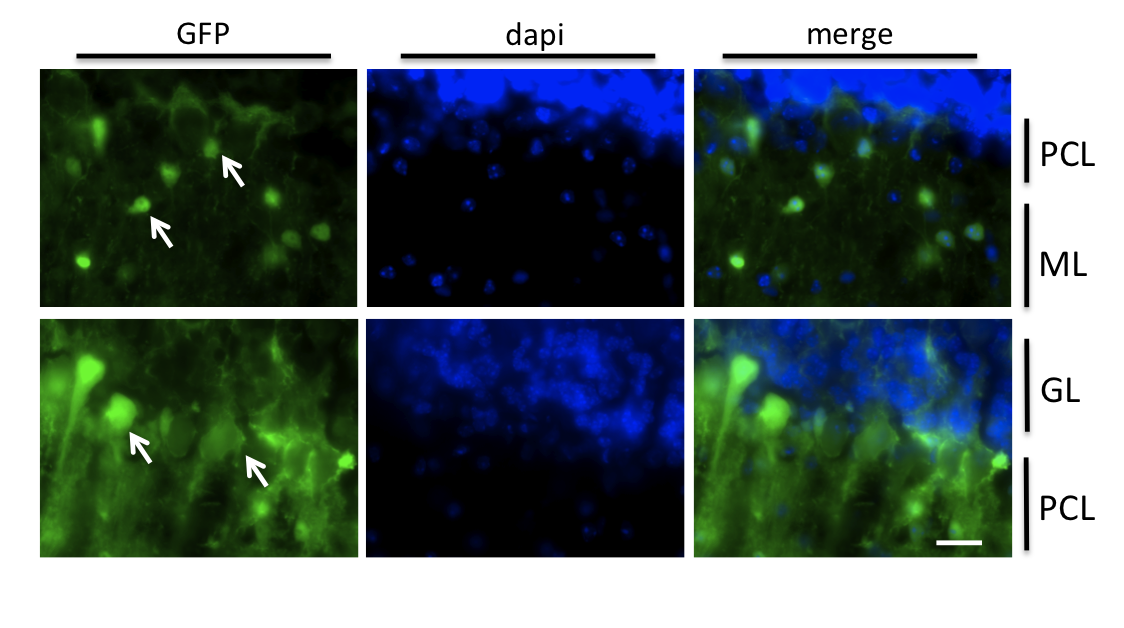

Supplement: Figure S3 — The intracerebellar injection of LV encoding for GFP in the cerebellar vermis mediates an extensive transduction of the cells in the cerebellar cortex, mainly the molecular layer cells (ML), and the Purkinje layer cells (PCL). (TIF) [file pone.0052396.s003.tif]

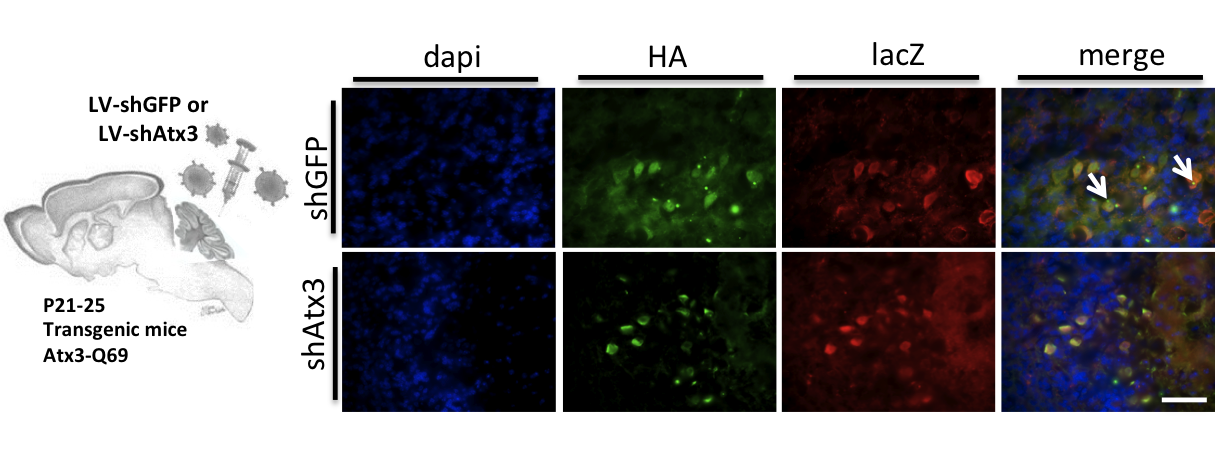

Supplement: Figure S4 — Transgenic mice injected with LV encoding a short-hairpin against GFP (shGFP) exhibit ataxin-3 aggregates (HA tag, green) co-localizing with LacZ (red, white arrows); whereas aggregates from mice injected with short-hairpins against mutant ataxin-3 (shAtx3) do not co-localize. This indicates that the shRNAs targeting mutant ataxin-3, but not the control shRNAs targeting GFP, prevent the formation of ataxin-3 aggregates. (TIF) [file pone.0052396.s004.tif]

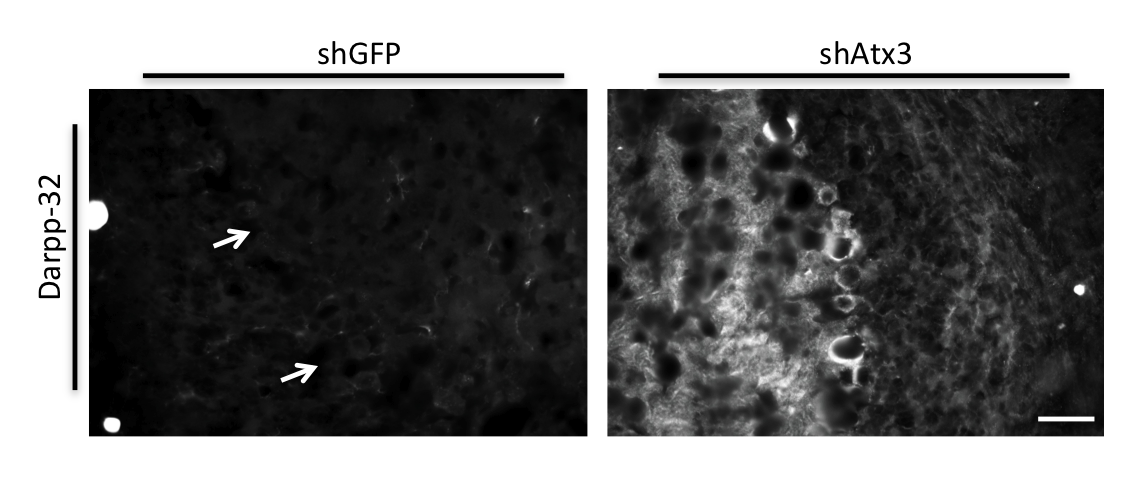

Supplement: Figure S5 — DARPP-32 staining revealed a preservation of immunoreactivity in transgenic mice injected with LV encoding shAtx3 (n = 8) as compared to mice injected with shGFP (arrows, n = 8). The figure shows representative images that were reproducible among the different groups of animals. Scale bar: 40 µm. (TIF) [file pone.0052396.s005.tif]

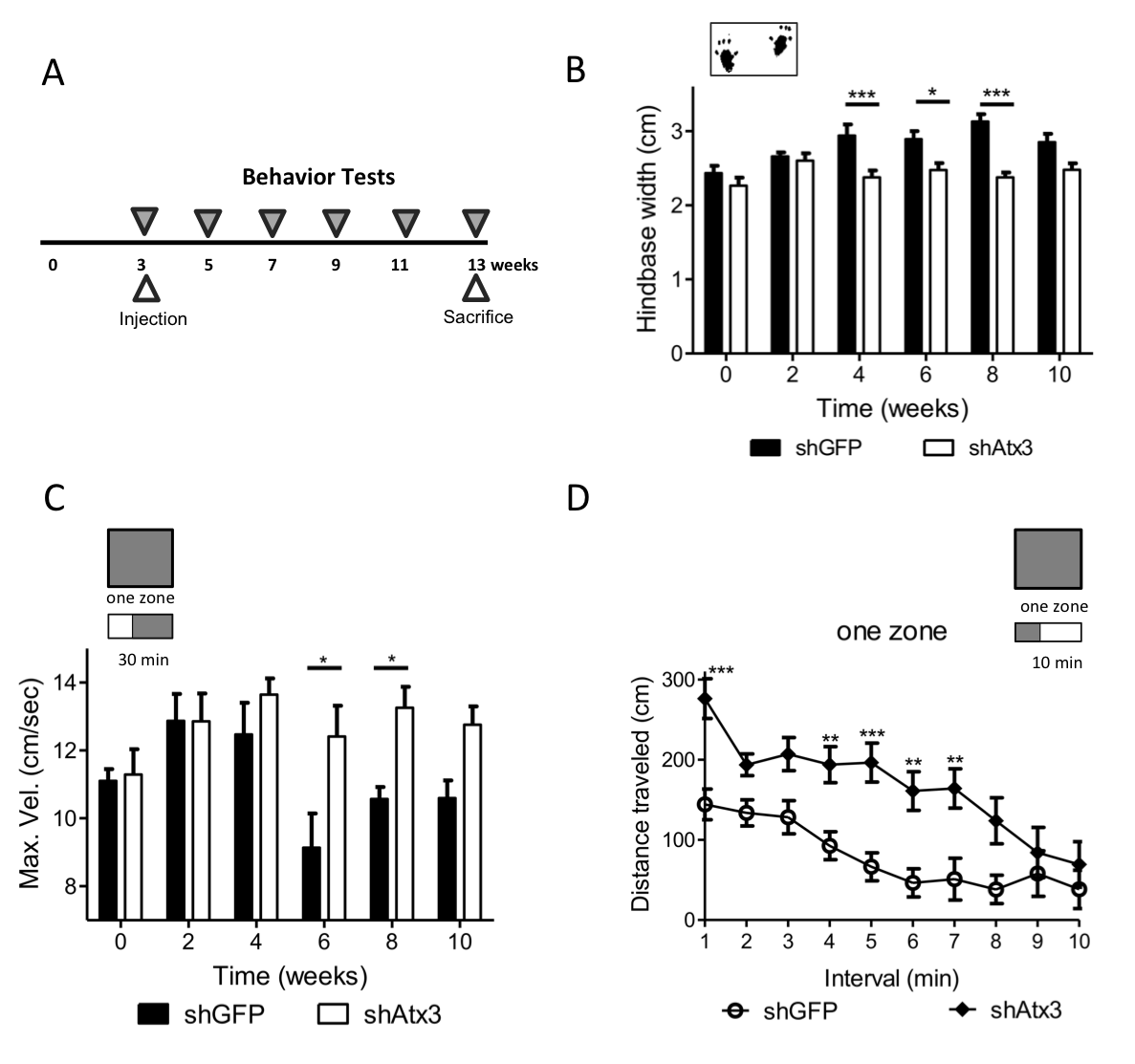

Supplement: Figure S6 — Behavior analysis of studied transgenic mice. A) Time course of mice behavior tests: rotarod performance test, footprints patterns analysis and activity box monitoring were assessed every 2 weeks post-injection until 10 weeks. B) Footprints patterns quantitative analysis. Hindbase width measures of shGFP injected mice (n = 8) show a significantly greater distance between left and right limb compared to shAtx3 injected mice (n = 8), indicating a higher deficit of coordination in control mice. *Statistical significance (*P<0.05; ***P<0.001; 2-way ANOVA, Bonferonni post-test). C) Locomotor horizontal activity of mice was tracked for 30 minutes (after a 10 minutes habituation period) and analyzed for maximum velocity (cm/sec). Mice injected with shAtx3 (n = 8) revealed a significantly better locomotor activity than control mice (shGFP, n = 8), as shown by faster movement from 4 weeks post-injection. *Statistical significance (*P<0.05; 2-way ANOVA, Bonferonni post-test). D) Allele specific silencing of mutant ataxin-3 improves exploratory activity. Analysis of the first 10 minutes in the cage at 10 weeks post-injection for one zone (arena not divided) revealed significantly-increased traveled distance in mice injected with shAtx3 (n = 8) compared to mice injected with shGFP (n = 8). (TIF) [file pone.0052396.s006.tif]
